# The association of advanced lung cancer inflammation index with non-alcoholic fatty liver disease in NHANES 2017–2020

**DOI:** 10.3389/fmed.2025.1516464

**Published:** 2025-04-14

**Authors:** Lin Cheng, Shumeng Li, Hui Li, Jiafeng You, Mingwei Yu, Guowang Yang

**Affiliations:** Beijing Hospital of Traditional Chinese Medicine Affiliated to Capital Medical University, Beijing, China

**Keywords:** non-alcoholic fatty liver disease, advanced lung cancer inflammation index, NHANES, cross-sectional study, positive correlation

## Abstract

**Background:**

The advanced lung cancer inflammation index (ALI) is a composite index that combines inflammation and nutritional status, and non-alcoholic fatty liver disease (NAFLD) is associated with inflammation, nutritional status, and obesity. This study aimed to investigate the possible relationship between ALI and NAFLD.

**Methods:**

We extracted cohort datasets from the 2017–2020 National Health and Nutrition Examination Survey (NHANES) for the study. Weighted analyses and multivariate linear regression models were applied to assess the association between ALI and NAFLD. Fitted curves and threshold effects analyses were used to characterize nonlinear relationships.

**Results:**

A total of 6,595 adults aged 18–80 years were included in this study. In multivariate linear regression analysis, there was a significant positive association between ALI and NAFLD [OR: 1.02, 95% CI (1.01, 1.02)]. In subgroup analyses, this positive association was maintained in females [OR: 1.02, 95% CI (1.01, 1.02)] and not in males. In addition, we found that the association between ALI and NAFLD was nonlinear, with an L-shaped relationship and an inflection point of 32.47. ALI showed a U-shaped association with NAFLD in the male population, with an inflection point of 40.65, and an L-shaped association in the female population, with an inflection point of 30.61.

**Conclusion:**

Our study suggests that there is a significant positive association between high ALI levels and NAFLD prevalence in the US adult population. However, more clinical cohort studies are needed to confirm this finding.

## 1 Introduction

About 30% of the global adult population suffers from non-alcoholic fatty liver disease (NAFLD), one of the most prevalent liver diseases in the world, with the greatest rate of morbidity and mortality among liver-related conditions (1). Except for severe alcohol use or other chronic liver illnesses, NAFLD is defined as steatosis in more than 5% of hepatocytes. The histologic changes in NAFLD include simple steatosis, nonalcoholic fatty liver (NAFL), and non-alcoholic steatohepatitis (NASH), which has a higher degree of inflammation and faster progression of fibrosis (2). In 2020, the European Liver Patient’s Association (ELPA) proposed replacing NAFLD with metabolic dysfunction-associated fatty liver disease (MAFLD), which is defined as hepatic fat deposition associated with obesity, diabetes mellitus, or mixed metabolic disorders (3). However, the change in terminology is hotly debated (4). While MAFLD may better align with metabolic risk factors, its more heterogeneous definition could potentially exclude patients with poorer prognoses (5, 6). Furthermore, since NAFLD better represents the underlying pathophysiological mechanisms of fatty liver disease (7), this study retains the use of the term “NAFLD.”

NAFLD progresses to progressive liver fibrosis and then to irreversible cirrhosis and even to hepatocellular carcinoma, and is becoming an important cause of end-stage liver disease (8). Liver biopsy is the gold standard for the diagnosis and grading of chronic hepatitis and liver fibrosis, however, liver biopsy is invasive, expensive, and has a high-risk factor (9). Therefore, the application of non-invasive indexes for early diagnosis and full management is the key to the prevention and treatment of NAFLD and related end-stage liver diseases. Previous studies have shown that obesity and being overweight are established risk factors for NAFLD (10). Inflammation is a key factor in the progression of liver fibrosis (11), which is driven by the production of inflammatory cytokines including IL-1, IL-6, TNF, and the infiltration of immune cells, such as macrophages, T-lymphocytes, and B-lymphocytes (2, 12, 13). A growing body of research has suggested that many nutritional/inflammatory variables can be valid predictors of NAFLD. For example, indicators of nutritional status, such as body mass index (BMI) and prognostic nutritional index (PNI), have been found to have a positive correlation with the risk of NAFLD (14, 15). The severity of NAFLD also positively correlates with inflammatory indicators, such as the systemic immune inflammation index (SII) and neutrophil-to-lymphocyte ratio (NLR) (16, 17). Therefore, assessing the combined effects of nutrition and inflammation may help to develop clinical intervention strategies to reduce the risk of NAFLD.

The ALI index consists of albumin (ALB), BMI, and NLR (ALI = BMI*ALB/NLR) (18). The ALI combines the nutrition-related index ALB, BMI, and the inflammation-related index NLR to provide a more comprehensive approach to identifying individuals with visceral obesity, metabolic abnormalities, and increased levels of body inflammation (19). The ALI is a comprehensive index that has emerged in recent years as a very promising marker for a variety of diseases including cancer and cardiovascular diseases (20–22). ALI serves as a valuable non-invasive biomarker for early NAFLD detection, offering significant potential in the comprehensive management of NAFLD and its associated end-stage liver complications through nutritional and inflammatory modulation. However, the relationship between ALI and NAFLD remains incompletely understood, so we analyzed extensive data from the 2017–2020 National Health and Nutrition Examination Survey (NHANES) for people aged 18 to 80 years to examine the relationship between ALI and NAFLD in adults.

## 2 Materials and methods

### 2.1 Survey description

To evaluate nutrition and health in the country, the National Center for Health Statistics (NCHS) administers the cross-sectional, population-based NHANES countrywide survey. Every NHANES research methodology was approved by the NCHS Research Ethics Review Board and Informed written consent was obtained from all participants before participation. The research design and data for the NHANES are fully detailed at www.cdc.gov/nchs/nhanes/.

### 2.2 Study population

**Figure 1:**
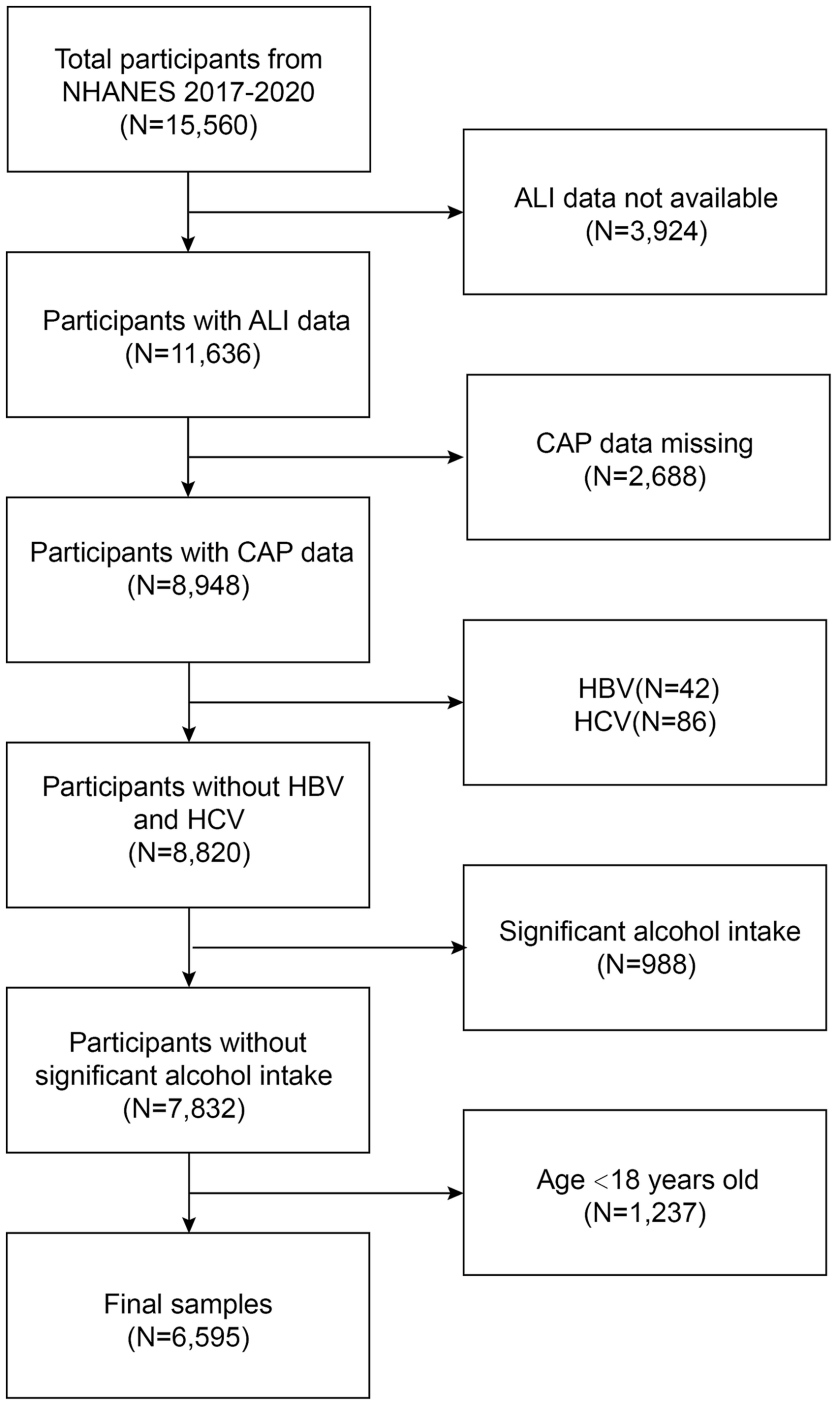
Flowchart of participant selection in NHANES 2017–2020. NHANES, National Health and Nutrition Examination Survey; ALI, advanced lung cancer inflammation index; CAP, controlled attenuation parameter.

This study used the US NHANES dataset for consecutive cycles from 2017 to 2020. 3,924 patients with missing ALI data were eliminated. 2,688 subjects with missing CAP data, 42 hepatitis B antigen-positive and 86 hepatitis C antibody-positive or hepatitis C RNA-positive samples, 988 subjects who drank large amounts of alcohol (4, 5, or more drinks per day), and 1,237 subjects under the age of 18 were excluded. In total, 6,595 participants were included in the study, out of 15,560 excluded as ineligible individuals (Figure 1).

### 2.3 Definition of ALI and NAFLD

The dependent variable in this study is the advanced lung cancer inflammation index, used to assess the patient’s nutritional and inflammation levels. Using the following formula, the ALI was calculated: BMI*ALB/ NLR. BMI was calculated in Kg/m^2^, and ALB was calculated in g/dL in the formula.

In this study, the Controlled Attenuation Parameter (CAP) was measured using Vibration controlled transient elastography (VCTE) to diagnose NAFLD in adults. CAP threshold ≥248 dB/m indicated the presence of hepatic steatosis in the participants, while a CAP value of less than 248 dB/m indicated the absence of a diagnosis of NAFLD (23).

### 2.4 Selection of covariates

In the current investigation, covariates included age, gender, race, education level, moderate actives status, smoking status, income-to-poverty ratio, BMI (Kg/m^2^), waist circumference (cm), total cholesterol (mg/dL), triglyceride (mg/dL), LDL-cholesterol (mg/dL), HDL-cholesterol (mg/dL), albumin (ALB, g/dL), alanine transaminase (ALT, U/L), aspartate aminotransferase (AST, U/L), alkaline phosphatase (ALP, IU/L), serum phosphorus (mg/dL), total calcium (mg/dL), and NLR.

### 2.5 Statistical analysis

Graphing software R (version: 4.1.3) and EmpowerStats (version: 4.2) were used to conduct the statistical study. The baseline table of the study population was statistically described by the presence or absence of NAFLD, and continuous variables were described using means plus or minus standard deviations (SDs) and weighted linear regression models. To examine the relationship between NAFLD and ALI, ALI levels were categorized into continuous and categorical variables (into quartiles, with the first quartile as the reference), and three models were constructed using multivariate tests: model 1: unadjusted for covariates, model 2: adjusted for gender, age, and race, and model 3: adjusted for all covariates. Multivariate linear regression analyses were performed to calculate odds ratios (ORs) and 95% confidence intervals (95% CI) for the three models. Interaction terms were also added to test for heterogeneity of associations between gender, age, BMI, and smoking status subgroups. Smoothed curve fitting was performed by adjusting for variables. The relationship and thresholds between ALI and NAFLD were tested using a threshold effects analysis model. Finally, the same statistical study methods described above were performed for the gender subgroups. *p* < 0.05 was considered statistically significant.

## 3 Results

### 3.1 Baseline characteristics of participants

A total of 6,595 adults were included in this study. The mean age of the participants was 48.51 ± 18.42, of which 45.19% were male and 54.81% were female. 12.54% were Mexican American, 34.16% were non-Hispanic White, 24.87% were non-Hispanic Black, and 28.43% were other races. The mean of the ALI was 33.78 ± 16.25. Compared to non-NAFLD participants, the NAFLD group was more likely to be male, older, with a larger proportion of non-Hispanic White, higher smoking status, and higher BMI, larger waist circumference, higher levels of total cholesterol, triglyceride, LDL-C, ALT, AST, ALP, ALI, triglyceride, higher levels of LDL-C, ALT, AST, ALP, ALI, and lower levels of education and HDL-C, ALB, serum phosphorus (Table 1).

**Table 1:** Basic characteristics of research objects.

Characteristics	Overall	Non-NAFLD	NAFLD	*p*-value
	*n* = 6,595	(CAP<274, *n* = 3,783)	(274 ≤ CAP, *n* = 2,812)	
Age (years)	48.51 ± 18.42	46.05 ± 19.21	51.82 ± 16.75	<0.001
Gender (%)				<0.001
Men	2,980 (45.19%)	1,565 (41.37%)	1,415 (50.32%)	
Women	3,615 (54.81%)	2,218 (58.63%)	1,397 (49.68%)	
Race (%)				<0.001
Mexican American	827 (12.54%)	364 (9.62%)	463 (16.47%)	
Non-Hispanic White	2,253 (34.16%)	1,260 (33.31%)	993 (35.31%)	
Non-Hispanic Black	1,640 (24.87%)	1,050 (27.76%)	590 (20.98%)	
Other Race	1875 (28.43%)	1,109 (29.32%)	766 (27.24%)	
Education level (%)				0.023
Less than high school	1,091 (17.48%)	575 (16.43%)	516 (18.81%)	
High school	1,436 (23.00%)	796 (22.74%)	640 (23.33%)	
More than high school	3,716 (59.52%)	2,129 (60.83%)	1,587 (57.86%)	
Moderate actives (%)				0.371
Yes	2,857 (43.32%)	1,621 (42.85%)	1,236 (43.95%)	
No	3,738 (56.68%)	2,162 (57.15%)	1,576 (56.05%)	
Smoked at least 100 cigarettes (%)				<0.001
Yes	2,284 (34.63%)	1,231 (32.54%)	1,053 (37.45%)	
No	4,311 (65.37%)	2,552 (67.46%)	1759 (62.55%)	
Income to poverty ratio	2.66 ± 1.64	2.65 ± 1.66	2.68 ± 1.61	0.426
BMI (Kg/m^2^)	29.83 ± 7.47	26.97 ± 5.94	33.68 ± 7.59	<0.001
Waist circumference (cm)	99.82 ± 17.18	92.34 ± 14.25	109.89 ± 15.59	<0.001
Total cholesterol (mg/dL)	184.90 ± 40.22	182.45 ± 39.75	188.20 ± 40.63	<0.001
Triglyceride (mg/dL)	108.09 ± 94.78	86.86 ± 64.50	136.84 ± 118.67	<0.001
LDL-cholesterol (mg/dL)	108.59 ± 35.17	106.49 ± 34.58	111.47 ± 35.78	<0.001
HDL-cholesterol (mg/dL)	53.40 ± 15.48	57.02 ± 15.45	48.54 ± 14.12	<0.001
ALB (g/dL)	4.08 ± 0.34	4.11 ± 0.34	4.04 ± 0.33	<0.001
ALT (U/L)	21.55 ± 16.43	18.18 ± 13.46	26.07 ± 18.82	<0.001
AST (U/L)	21.23 ± 12.14	20.19 ± 10.64	22.63 ± 13.79	<0.001
ALP (IU/L)	77.36 ± 24.56	74.79 ± 24.36	80.81 ± 24.41	<0.001
Serum phosphorus (mg/dL)	3.57 ± 0.52	3.60 ± 0.51	3.54 ± 0.52	<0.001
Total calcium (mg/dL)	9.28 ± 0.38	9.28 ± 0.37	9.28 ± 0.39	0.061
NLR	2.06 ± 1.11	2.06 ± 1.14	2.08 ± 1.08	0.01
ALI	33.78 ± 16.25	32.65 ± 16.80	35.29 ± 15.36	<0.001

Mean ± SD for continuous variables: *p* value was calculated by weighted linear regression model.

% for categorical variables: *p* value was calculated by weighted chi-square test.

BMI, body mass index; LDL-cholesterol, low-density Lipoprotein Cholesterol; HDL-cholesterol, high-Density Lipoprotein Cholesterol; ALB, Albumin; ALT, alanine transaminase; AST, aspartate aminotransferase ALP, alkaline phosphatase; NLR: neutrophil-to-lymphocyte ratio; CAP, controlled attenuation parameter; ALI, advanced lung cancer inflammation index.

### 3.2 The association between ALI and NAFLD

Table 2 provided further insight into the association between ALI and NAFLD through weighted multifactor logistic regression analysis. Continuous variable analysis of ALI showed that ALI and NAFLD were highly correlated in unadjusted models 1 and 2 (adjusted partially). In contrast, in model 3, the relationship between ALI and NAFLD became negative (adjusted for all covariates), although this relationship was not significant. Further analysis of quartiles of ALI showed a significant positive association between higher ALI and NAFLD in un-adjusted model 1 and partially adjusted model 2. In model 2, participants in the highest ALI quartile had a significantly higher risk of NAFLD by 1.16-fold compared with participants in the lowest ALI quartile [OR = 2.16, 95% CI (1.86–2.51), *p* < 0.0001].

**Table 2:** The association between ALI and CAP.

Variable	Model 1		Model 2		Model 3	
	OR (95%CI)	*p*	OR (95%CI)	*p*	OR (95%CI)	*p*
ALI (Continuous variables)	1.01 (1.01, 1.01)	<0.0001	1.02 (1.01, 1.02)	<0.0001	0.99 (0.99, 1.00)	0.1895
Stratified by ALI quartiles						
Q1 (3.73–23.38)	Ref		Ref		Ref	
Q2 (23.39–30.40)	1.44 (1.25, 1.66)	<0.0001	1.41 (1.22, 1.64)	<0.0001	1.14 (0.78, 1.67)	0.5075
Q3 (30.41–40.21)	1.84 (1.59, 2.11)	<0.0001	1.87 (1.62, 2.16)	<0.0001	1.42 (0.89, 2.27)	0.1371
Q4 (40.21–398.20)	1.88 (1.63, 2.17)	<0.0001	2.16 (1.86, 2.51)	<0.0001	1.30 (0.72, 2.37)	0.3847
P for trend	<0.0001		<0.0001		0.3401	

Model 1: no covariates were adjusted. Model 2: age, gender, and race were adjusted. Model 3: age, gender, race, educational level, moderate actives, smoking status, income-to-poverty ratio, moderate activities, BMI, waist circumference, total cholesterol, triglyceride, LDL-cholesterol, HDL-cholesterol, ALB, ALT, AST, ALP, serum phosphorus, total calcium, and NLR were adjusted.

### 3.3 Subgroup analysis

Participants were analysed in model 2 (adjusted partially) stratified by gender, age, BMI, and smoking status to further explore the relationship between ALI and NAFLD. The results showed that gender stratification significantly altered the relationship between ALI and NAFLD (*p* = 0.0035) (Table 3).

**Table 3:** Association between ALI and NAFLD by selected subgroups.

Subgroup	OR (95%CI) *p* value	P for interaction
Gender		0.0035
Male	1.01 (1.00, 1.01) <0.0001	
Female	1.02 (1.01, 1.02) <0.0001	
Age (years)		0.1508
Age < 60	1.02 (1.01, 1.02) <0.0001	
Age ≥ 60	1.01 (1.01, 1.02) <0.0001	
BMI (Kg/m^2^)		0.3447
BMI < 25	1.01 (1.00, 1.02) 0.1727	
25 ≤ BMI < 30	1.00 (0.99, 1.00) 0.5798	
BMI ≥ 30	1.00 (1.00, 1.01) 0.5058	
Smoked at least 100 cigarettes		0.4126
Yes	1.02 (1.01, 1.02) <0.0001	
No	1.01 (1.01, 1.02) <0.0001	

### 3.4 Nonlinear relationship and threshold effect analysis between ALI and NAFLD

**Figure 2:**
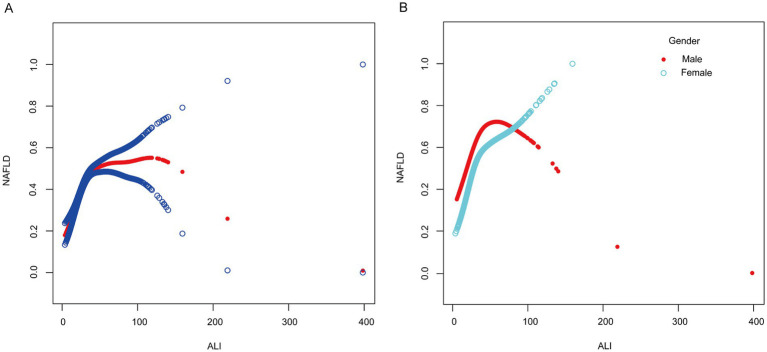
Smooth curve fitting between ALI and NAFLD. **(A)** The solid red line represents the smooth curve fit between the overall population variables. Blue bands represent the 95% confidence interval from the fit. **(B)** The red and blue bands, respectively, represent smooth curve fits between ALI and NAFLD in males and females.

Smoothed curve fitting was used to investigate the nonlinear relationship between ALI and NAFLD in order to identify any threshold or saturation effects. The results showed a nonlinear relationship between ALI and NAFLD [OR = 1.02, 95% CI (1.01–1.02), *p* < 0.0001] (Table 4). Threshold effect analysis showed that at 32.47, the positive correlation between ALI and NAFLD changed, showing an L-shaped curve. After stratifying the analysis by gender, females still had an L-shaped curve, with higher levels of ALI positively associated with the risk of NAFLD prevalence. A U-shaped curve existed in males, with a negative association between ALI and NAFLD when ALI was >40.65 (Figure 2; Table 4).

**Table 4:** Analysis of threshold effect and saturation effect.

ALI	Overall	Male	Female
Model I			
A straight-line effect OR (95%CI) *p* value	1.02 (1.01, 1.02) <0.0001	1.01 (1.01, 1.02) <0.0001	1.02 (1.01, 1.02) <0.0001
Model II			
Fold points (K)	32.47	40.65	30.61
<K-segment effect 1 OR (95%CI) *p* value	1.05 (1.04, 1.06) <0.0001	1.04 (1.03, 1.05) <0.0001	1.06 (1.04, 1.07) <0.0001
>K-segment Effect 2 OR (95%CI) *p* value	1.00 (1.00, 1.01) 0.2828	1.00 (0.99, 1.00) 0.2755	1.01 (1.00, 1.01) 0.0653
Log likelihood ratio tests	<0.001	<0.001	<0.001

Age, gender, and race were adjusted.

## 4 Discussion

In this cross-sectional study involving 6,595 adults, a positive association between high ALI and the risk of NAFLD was observed. This association remained significant even after adjusting for gender, age, and race. Findings showed that each one-unit increase in ALI was associated with a 2% increase in the risk of NAFLD prevalence [OR: 1.02, 95% CI (1.01, 1.02)], and participants in the highest quartile of ALI had a 1.16-fold increase in the risk of NAFLD compared with the lowest quartile (*p* < 0.001).

The advanced lung cancer inflammation index was calculated as BMI*ALB/NLR. The concept of ALI was first introduced in 2013 to predict survival and inflammation levels in patients with metastatic non-small cell lung cancer (24). Subsequent studies have shown that ALI can significantly predict the prognosis of malignant diseases such as colorectal, gastric, and liver cancers (25–28). A previous study collected clinical data on 1,654 postoperative patients and found that higher ALI levels were associated with improved 1-, 3-, and 5-year survival rates, highlighting its utility in predicting post-hepatectomy outcomes (21). Beyond its applications in oncology, ALI has also shown significant predictive value for cardiovascular diseases and chronic inflammatory conditions such as rheumatoid arthritis (29, 30).

The pathogenesis of NAFLD remains incompletely understood. One prominent theory posits that overnutrition drives fat accumulation in adipose tissue and ectopic sites, triggering macrophage infiltration, chronic inflammation, and insulin resistance, which disrupts the balance between lipid catabolism and synthesis (31). This metabolic dysfunction may lead to lipotoxicity, oxidative stress, and cell death (32, 33), as well as the subsequent fibrotic process (34). Another idea contends that liver-organ connections and dependencies may cause liver metabolism to become dysregulated, which would increase inflammation and aid in the progression of NAFLD (35–37). Some data suggest that gut microorganisms are significantly altered in patients with NAFLD compared to normal subjects, with a reversal of the ratio of Thickettsia to Mycobacterium and an increase in Aspergillus phylum (38). Meanwhile, bile acids play an important role in regulating the gut microbiome, which may have an impact on the degree of hepatic inflammation and fibrotic progression in NAFLD (39), however, more studies are needed to clarify this conclusion.

Our findings suggest that ALI is positively associated with NAFLD prevalence, potentially due to its components: NLR, ALB, and BMI. Elevated NLR reflects systemic inflammation, which is common in NAFLD and its progression to NASH (40, 41). A cross-sectional study of 1,620 U.S. adults also linked higher NLR to increased liver fibrosis scores in NAFLD patients, consistent with our results (42). Additionally, ALB is a key marker of nutritional status and a major prognostic factor in liver disease (43). In patients with advanced cirrhosis, ALB levels decrease by 60–80%, accompanied by multiple post-translational modifications that alter its structure and functional properties (44). Our study found significantly lower ALB levels in NAFLD participants compared to the normal group (*p* < 0.001). From the above findings, we can infer that the ratio of ALB to NLR was reduced in NAFLD participants. Finally, numerous studies have demonstrated that higher BMI, particularly visceral obesity, contributes to hepatic fat accumulation (45). In obesity, the expansion of white adipose tissue, particularly visceral fat, results in the release of excess non-esterified fatty acids into the portal circulation. According to the portal vein theory, this process drives hepatic lipid accumulation and accelerates the development of steatosis (46). This is consistent with our findings that NAFLD participants had higher BMI values compared to non-NAFLD participants. In light of the aforementioned, we think that BMI is more important in the favorable correlation between ALI and NAFLD.

Our study reveals for the first time that there are gender differences in the association between ALI and NAFLD. In the adult male population, the association between ALI and NAFLD exhibited an inverted U-shaped relationship, with a positive correlation below an ALI threshold of 40.65 and a negative correlation above it. In contrast, females displayed an L-shaped relationship, where ALI was positively associated with NAFLD prevalence below a threshold of 30.61. Above this threshold, the association remained positive but was not statistically significant (*p* = 0.0653). The results of this study may be related to the secretion of sex hormones. Several studies have shown that the prevalence of NAFLD is reduced in premenopausal females compared to males, while postmenopausal females exhibit similar rates to age-matched males (47, 48). This is similar to our findings that the male population was more predominant in NAFLD participants compared to the non-NAFLD population (*p* < 0.001), suggesting a protective effect of estrogen. In a previous study, it was shown that estrogen enhances the insulin sensitivity of adipose tissue and reduces adipose tissue catabolism (49). Another study highlighted that estrogen-related receptor alpha regulates the production and secretion of triglyceride-rich very low-density lipoproteins in the liver, preventing excessive accumulation of lipids in the liver and reducing hepatic inflammatory injury (50). In addition, fat distribution may also be another factor for gender differences. In men, adipose is more distributed in the abdomen and viscera, while in women, adipose is more distributed in the hips and lower limbs (51). Compared to abdominal adipose tissue, gluteal and lower limb adipose have a lower lipolytic response to epinephrine and norepinephrine, reducing free fatty acid transport to the liver (52). Although the definitive mechanism for this gender difference is not fully understood, current evidence indicates that persistently elevated ALI levels in women are linked to a higher prevalence of NAFLD and warrant close monitoring. However, in the male population, identifying an ALI inflection point is crucial, as NAFLD prevalence may decline beyond a specific ALI threshold. The clinical treatment and non-invasive prediction of NAFLD may benefit from these insights.

This study has several strengths. First, our research is based on a nationally representative sample of the multi-ethnic diverse adult population in the United States. Second, to our knowledge, while one study initially demonstrated an association between ALI and NAFLD (53), further research in this area remains limited. Having confirmed the positive association, we expanded the scope of investigation through subgroup analyses and threshold effect analyses to fill the relevant research gaps. Additionally, as a composite index combining inflammation and nutritional markers, ALI shows potential as a non-invasive biomarker for NAFLD, aligning with the need for accessible diagnostic tools in hepatology. However, this study still has some limitations. First, this was a cross-sectional study and therefore we were unable to determine a causal relationship between ALI and NAFLD. Second, hepatic steatosis in this study was assessed using VCTE. Although current studies have demonstrated its high accuracy and sensitivity, it remains inferior to the gold standard of liver biopsy (54, 55). Third, while ALI combines BMI, ALB, and NLR, it may not fully capture the multifactorial nature of NAFLD pathogenesis, which involves complex metabolic, inflammatory, and genetic interactions (56). Fourth, due to the incomplete data records on women’s menopause within the NHANES database for 2017–2020, this study was unable to evaluate the potential impact of pre- and post-menopausal age differences on the observed nonlinear associations in the female population. Finally, the U-shaped and L-shaped nonlinear associations identified in this study may increase the complexity of clinical interpretation. Therefore, further prospective studies with larger samples are warranted to validate these findings.

## 5 Conclusion

This cross-sectional study suggests that there is a significant positive association between high ALI levels and the prevalence of NAFLD in the US adult population. However, more clinical cohort studies are needed to confirm this finding.

## Data Availability

The datasets presented in this study can be found in online repositories. The names of the repository/repositories and accession number(s) can be found in the article/supplementary material.
